# Autofluorescence Image-Guided Endoscopy in the Management of Upper Aerodigestive Tract Tumors

**DOI:** 10.3390/ijerph20010159

**Published:** 2022-12-22

**Authors:** Norhafiza Mat Lazim, Abdul Hafeez Kandhro, Anna Menegaldo, Giacomo Spinato, Barbara Verro, Baharudin Abdullah

**Affiliations:** 1 Department of Otorhinolaryngology-Head and Neck Surgery, School of Medical Sciences, Universiti Sains Malaysia, Health Campus, Kubang Kerian 16150, Malaysia; 2 Institute of Medical Technology, Jinnah Sindh Medical University, Karachi 75510, Pakistan; 3 Department of Neurosciences, Section of Otolaryngology and Regional Centre for Head and Neck Cancer, University of Padova, 31100 Treviso, Italy; 4 Department of Surgery, Oncology and Gastroenterology, Section of Oncology and Immunology, University of Padova, 31100 Treviso, Italy; 5 Division of Otorhinolaryngology, Department of Biomedicine, Neuroscience and Advanced Diagnostic, University of Palermo, 90127 Palermo, Italy

**Keywords:** head and neck cancers, autofluorescence, narrow band imaging, surgical margins, endoscopy, upper aerodigestive tract tumor, oral cavity carcinoma, squamous cell carcinoma

## Abstract

At this juncture, autofluorescence and narrow-band imaging have resurfaced in the medicine arena in parallel with current technology advancement. The emergence of newly developed optical instrumentation in addition to the discovery of new fluorescence biomolecules have contributed to a refined management of diseases and tumors, especially in the management of upper aerodigestive tract tumors. The advancement in multispectral imaging and micro-endoscopy has also escalated the trends further in the setting of the management of this tumor, in order to gain not only the best treatment outcomes but also facilitate early tumor diagnosis. This includes the usage of autofluorescence endoscopy for screening, diagnosis and treatment of this tumor. This is crucial, as microtumoral deposit at the periphery of the gross tumor can be only assessed via an enhanced endoscopy and even more precisely with autofluorescence endoscopic techniques. Overall, with this new technique, optimum management can be achieved for these patients. Hence, the treatment outcomes can be improved and patients are able to attain better prognosis and survival.

## 1. Introduction

Head and neck tumors represent 5% of all neoplasms worldwide and manifest differently based on the patient demographic (e.g., HPV status) and the anatomical site which harbors the neoplasm (e.g., oral tongue versus tonsils). The majority of head and neck tumors are squamous cell carcinoma, which mostly arise in the upper aerodigestive tract mucosa, namely the oral cavity, pharynx, larynx, nasal cavity, paranasal sinus and nasopharynx [1]. The majority of upper aerodigestive tract tumor patients present at late stage, where the treatment is more challenging. This is compounded by multiple patient factors, as well as logistic factors that affect the treatment outcomes. Most patients who come with late-stage tumors have poor prognosis, especially in the presence of bulky tumors and distant metastasis. Despite poor prognosis, early screening, early treatment and a well-planned follow up scheme can lead to overall improved survival and quality of life of these patients.

Generally, for head and neck malignancy, a significant number of patients presented with neck recurrence, as well as multiple systemic metastases, despite multimodality treatment, whereas a minority of patients had second primaries. The contributing factors for these poor outcomes include late diagnosis during a primary tumor assessment and characteristic tumor biology, as well as a high prevalence of second primary tumors and recurrent tumors in patients who already have received treatment for head and neck carcinoma [2,3]. In addition, the prognosis is also strongly related to early screening, availability of accurate diagnostic procedures and adequate surgical resection margin of any given tumors [4,5,6]. This is vital in order to improve patient treatment outcomes and prognosis. A wide availability of effective screening methods and up-to-date and highly sensitive diagnostic procedures will ultimately improve head and neck carcinoma patients’ subsequent management.

Of note, in parallel with recent advancement in technology, the latest imaging techniques which are commonly applied for detecting cancer in any head and neck surgical oncology center are based on the structural changes in the tissues. In addition, advancement in optical appliances and hardware, nanomolecules and reagents has provided ideal opportunities for cancer screening and diagnosis, either in the clinic, ward or operating room settings. In addition, nowadays, patients also seek less invasive and less time-consuming procedures. The quest for a safe, efficient and less time-consuming diagnostic method capable of detecting premalignant and malignant lesions has led to the generation of photodiagnosis by autofluorescence (AF) endoscopy techniques.

Autofluorescence is the fluorescence that is naturally emitted by the primary fluorophores within specific tissues. The endogenous fluorophores include collagen with its nicotinamide adenine dinucleotide dehydrogenase (NADH) and flavin adenine dinucleotide (FAD), metabolic products and other structural proteins which are the content of the tissues [7]. These endogenous fluorophores absorb light at specific wavelengths and correspondingly emit light at a longer wavelength (lower energy), which is referred to as autofluorescence. Fluorescence lifetime is considered a state function as it does not depend on initial perturbation conditions, such as duration of light exposure, excitation wavelength, fluorophore concentration and photobleaching, and is independent of fluorescence intensity [8].

**Figure 1:**
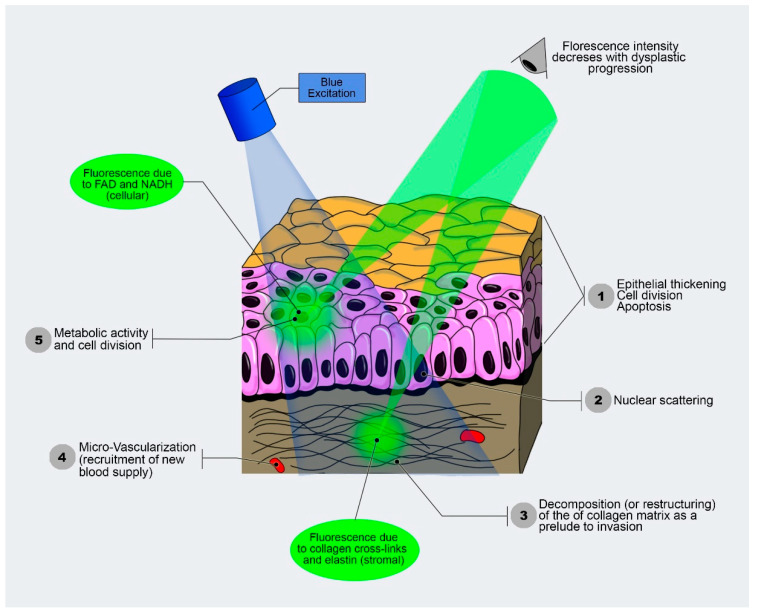
Autofluorescence functioning based on different fluorescence characteristics of the tissues, which illuminates with different intensity (modified from Optical Imaging Lab-screening and diagnosis with light, Vancouver, Canada).

These fluorophores can be a good indication of the pathological state of the tissues, as different tissues show different fluorescence [9]. Imperatively, the tumor cells are also able to produce a higher intensity of fluorescence and, depending on their contents of (NADH and FAD), they have a longer fluorescence lifetime, which can be applied in cancer screening and diagnosis. Generally, in the tumor, there is neoangiogenesis and hypervascularization. This can be associated with a relatively high blood content of hemoglobin. The hemoglobin contains the NADH and FAD. The fluorescence of NADH and FAD may increase due to the Warburg effect, or as cells become dysplastic due to the disruption of the extracellular matrix during tumor progression [10,11]. Since alterations in the blood supply and in the architectural organization result in a noticeably enhanced cellularity in the tumor mass, the involvement of tissue optical characteristics is crucial because they alter the scatter phenomena, absorption, and reflectance, thus impacting the movement of light. This also results in excitation and fluorescence collection from various tissue depths (Figure 1). Indeed, the expansion of the neoplastic mass affects the integrity and depth of localization of the highly fluorescing submucosa [12].

Autofluorescence imaging (AFI) is an imaging modality that helps to visualize the autofluorescence bands of the endogenous fluorophores. When a specific wavelength of light and narrow-band filter are used, the normal mucosa appears as pale green autofluorescent compared with neoplastic tissues, which are visualized as a darker area due to the autofluorescence loss [7,13,14]. This is mainly due to loss of fluorophore collagen. Malignant tissues are characterized by numerous architectural changes such as high nuclear grade and mitochondrial density, as well as different amounts of keratin, elastin and collagen, which contribute to the different spectral characteristics if compared with the normal tissues [15]. These optical techniques such as microendoscopy and spectroscopy have been widely used to detect premalignant and malignant lesions based on subtle surface changes associated with mucosal ulceration and growth. These strategies have also been applied in a surgical setting, where the parameters of the tumor can be delineated effectively using optical imaging [13]. This ensures a complete resection of the tumor with a clear surgical margin. This clear surgical margin is vital in oncologic surgery, as significant recurrent diseases and metastatic deposits are likely to occur if the surgical margins are involved.

## 2. Autofluorescence and Imaging

Tissue autofluorescence and its potential application in the screening and diagnosis of cancer were first described in the early 1900s. The autofluorescence technique is a phenomenon where the endogenous fluorophores are excited by an extrinsic light source. The endogenous fluorophores such as certain amino acids, metabolic products and structural proteins absorb photons from the exogenous light source and emit lower-energy photons. These lower-energy photons present clinically as fluorescence. Each fluorophore is associated with specific excitation and emission wavelengths. Of note, when irradiated with light of wavelengths between 375 and 440 nm, the fluorochromes show fluorescence in the green spectrum. In contrast, when viewed through a selective narrow-band filter, an unaltered healthy mucosa yields a lighter green autofluorescence. 

Imperatively, the autofluorescence imaging works on the assumption that structural changes or abnormal metabolic changes in the pre-neoplastic and neoplastic tissues have reflectance and absorbance that is varied when exposed to specific wavelengths of lights [16]. The spectrum of fluorescence of the ultraviolet (UV) and visible region of the tissue is mainly due to the presence of the connective tissue’s collagen. In the epithelium, keratin is also able to produces fluorescence. However, due to the presence of mitochondrial NADH and FAD in the basal cells of the epithelium, epithelium always shows weaker autofluorescence. As far as tumors are concern, increased mitochondrial fluorescence of the epithelium is observed in conditions such as epithelial dysplasia. Importantly, loss of autofluorescence observed in neoplasia is due to the loss of stromal collagen. In addition, in the case of inflammatory lesions, loss of both stromal and epithelial autofluorescence can also be observed [17].

There are many autofluorescence imaging (AFI) techniques available for the detection of carcinogenesis and malignant transformations in the current market (Table 1). These systems include the DAFE system (Richard Wolf, Knittlingen, Germany), the SAFE system (Pentax, Tokyo, Japan), the LIFE system (Xillix Technology, Montreal, QC, Canada) and the D-Light-AF system (Karl Storz, Tuttlingen, Germany). These systems are characteristically designed for usage at the other areas but can be applied to the head and neck region, as the principles of application and detection are similar. For oral cavity inspection, AFI systems are specifically designed, such as the VELscope LED Medical Diagnostics (Vancouver, BC, Canada) and Identafi DentalEZ, (Lancaster, PA, USA) [13]. Other AFI systems include ViziLite, ViziLite Plus and MicroLuxTM/DL [16].

A VELscope is a simple camera device which can be handheld during a procedure. It is noninvasive, convenient to use and capable of visualizing and documenting tissue autofluorescence alterations, for instance, in the oral cavity, oropharynx and sinonasal cavity [14,22,23]. For the usage of VELscope, there is no special training necessary at both the general and subspecialty practices. This device emits blue light (400 and 460 nm wavelength) for excitation of endogenous fluorophores [24]. The healthy tissue after illumination gives pale green appearances, whereas the abnormal tissue exhibits loss of autofluorescence and is observed as dark areas in contrast to the surrounding normal tissue. The VELscope is applicable for the screening of abnormalities, such as identifying neoplasm margins and the detection of neoplastic transformation [13,24].

On the other hand, Identafi is more reliable than VELscope. It has multimodality imaging, which provides three kinds of lights, including white light, green light and violet light. For conventional oral examination, white light is used, while the other violet and green lights are used to facilitate the examinations. The violet light, which is 405 nm wavelengths of light, is able to differentiate the malignant tissue from normal tissue mucosa based on the autofluorescence loss, which is similar to a VELscope. While, in the case of narrow-band imaging (NBI), the peaks of absorption wavelengths of the green–amber light with 545 nm wavelengths matches that of hemoglobin [13]. This is useful for the assessment of neoangiogenesis, which is a critical feature of malignant mass. 

In the D-LIGHT C/AF System (Karl Storz, Germany), a Xenon arc lamp is incorporated with the blue light source, which has a wavelength of 375 to 440 nm. In this real-time system, the white-light endoscopy can be efficiently changed to the autofluorescence-based endoscopy. An integrated optic filter is used to block out the remitted excitation light of up to 440 nm. A limitation of this system is that it is capable of detecting changes on laryngeal epithelium but unable to detect changes in deeper structures. Due to this reason, laryngeal nodule, Reinke’s edema, polyp, papilloma and other lesions do not yield any disturbances in the autofluorescent signals. Importantly, if autofluorescent intensity is reduced, it is important to consider a particular area as a suspicious and to take a biopsy [25].

ViziLiteR (United States) is a light-based device which use chemical agents: hydrogen peroxide and acetylsalicylic acid in a disposable capsule. It is used for early identification of potentially malignant lesions and oral cavity squamous cell carcinoma (OCSCC) [26]. ViziLiteR is the first device approved by the FDA in 2002. The capsule is made from a flexible plastic outer shell, in which for activation the capsule needs to be bent to break the inner glass. This will trigger a reaction of the chemical content and, as a result, a bluish light of 430–580 nm wavelength is produced. In a study by Mehrotra et al., in which ViziLiteR and VELscope were compared to evaluate their applications in screening for suspicious oral malignant lesions, the result, however, was comparable to the conventional oral examination technique. Due to this reason, a new and improved device (ViziLiteR PLUS) has been made available in the market in order to improve its diagnostic power [27].

A proper filtration is necessary because high-intensity light is used for fluorochromes excitation. The pale and narrow autofluorescence signal will not appear without a proper filtration. The dysplastic tissues appear darker due to a disruption in the distribution of the fluorochromes in comparison with the surrounding normal tissue [26]. Other endogenous fluorophores have to be accounted for when analyzing the appearance of the autofluorescence signal. For instance, the connective tissue of the oropharynx has a remarkably reduced fluorescence intensity in comparison with the larynx. The elastic fibers of the lamina propria of laryngeal and oral mucosa have a high fluorescent intensity. This justifies the disappearance of bright green fluorescence in fibrotic laryngeal mucosa due to the replacement of thin elastic fibers by less fluorescent collagen fibers during the healing process. NADH is a strong endogenous fluorophore which, in malignant tissues, its nonfluorescent oxidated form dominates, and consequently the intensity of fluorescence is reduced [28].

Considerable literature reported that different fluorophores can be the source of AF, for instance, collagen, elastin, flavins, NADH and keratin. Thus, a sensitive bioindicator for monitoring cell metabolism may be comprised of any coenzyme fluorescence in the blue–green spectrum. Noteworthily, the AF can be influenced by many factors such as hemoglobin absorption, blood flow, tissue structures and scattering of the applicated light [29]. This needs to be fully considered when interpreting the AF results based on the characteristics of the tissues and organs examined.

## 3. Clinical Application of Autofluorescence in Cancer Management

Recently, the application of autofluorescence endoscopy and imaging in biomedicine has surged globally in ensuring the optimum management of diseases and tumors, inclusive of head and neck malignancies. This applied new technology translates to better prognosis, survival and quality of life of any treated patients with upper aerodigestive tract tumors. For instance, early screening of malignant changes in the upper aerodigestive tract mucosa is the best strategy to enhance patient survival. Thus, screening is vital, especially targeting the population at high risk in certain geographic locations. Recently, many techniques that are frequently used in the diagnosis of oral cavity cancers, namely toluidine blue, Lugol’s iodine and brush biopsy, have dwindled due to certain limitations. In addition, the recent method of visual examination of the oral cavity for diagnosis of malignancy depends on the experience of the clinician in identifying early malignant changes. This requires a highly skilled and experienced surgeon and clinician who are able to detect suspicious tumor lesions during initial consultation. Indeed, recognizing the premalignant and early malignant lesions from benign inflammatory diseases by direct visual examination is challenging, not only for the trainee clinician but also for experienced practitioners.

Imperatively, the premalignant lesions showed variable AF appearance. For example, mucosal alterations, which are characterized by overproduction of keratin or hyperkeratosis, are challenging to be recognized as harboring malignant cells. Macroscopically, in the majority of cases, these lesions appear as whitish grey “spots” on the surface of unhealthy mucosa. Generally, they exhibit a high fluorescence intensity and appear as a light green which is lighter than the healthy surrounding mucosa. Importantly, these lesions can mimic benign pathology in case there are no other characteristic fluorescence that can assist in identifying the underlying malignant transformation, such as the presence of violet or reddish blue spots [30]. Jo et al. reported that early dysplasia and early oral cancer can be distinguished from benign lesions by using multispectral endogenous fluorescence lifetime imaging [31]. This is based on a diagnostic computer generation system derived from the biochemical and biomarker used. VELscope is a nonmagnifying device, which is used for direct visualization of oral mucosa. In this device, an arc lamp of 120 W and a series of filters and reflectors were optimized for producing a 400–460nm wavelength light. In the oral mucosa, emitted light reaches and excites endogenous autofluorescence substances, known as fluorophores. This produces the autofluorescence signals, which are tissue-specific. It can be one of the selected devices used to further assess the hyperkeratotic lesions of the head and neck region.

A study by Mascitti et al. comprised of 44 patients with oral dysplasia and OCSCC using both conventional oral examination and VELscope. They found out that the device gives high sensitivity and specificity for the differentiation of potentially malignant disorders and OCSCC from the normal healthy oral mucosa [19,32]. This is a vital study which highlight the effective usage of AF devices. Future studies are required to improve the specificity of this device, which may allow VELscope to be used in wider clinical applications in routine general practice globally [19,33]. Detection of precancerous lesions is challenging due to the multistep processes involved, in addition to field cancerization. Usually, direct visualization and inspection is used to diagnose early malignant lesion of the upper aerodigestive tract. This essentially depends on the expertise of the in-charged clinician in recognizing any suspicious lesions during clinical examination. However, it is challenging to detect and distinguish early malignant lesions from benign inflammatory pathology during a conventional white-light endoscopy, as this lesion may appear as normal healthy mucosa [30].

### 3.1. Screening of Aerodigestive Tract Tumors by Autofluorescence

**Figure 2:**
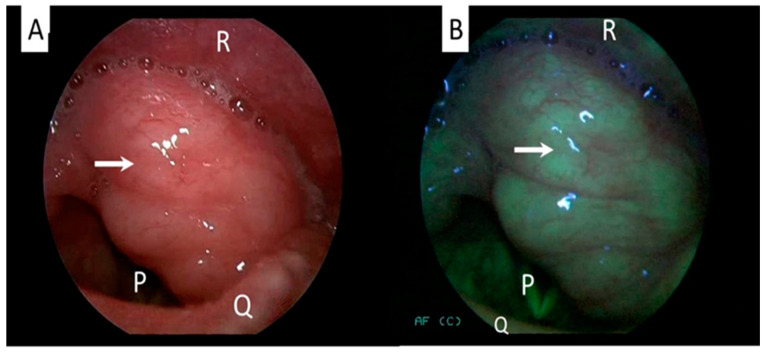
Laryngeal examination of a patient with benign laryngeal tumor showing no changes in the mucosa between white-light (**A**) and autofluorescence mode (**B**). Arrow indicates the tumor. Structure P: vocal cord/glottic, Q: epiglottis, R: posterior pharyngeal wall (Figure is from author’s own collection).

The screening and early detection of tumors are vital for achieving higher curative rates and improving prognosis of head and neck cancer patients. Oral cavity carcinoma is a significant tumor of the head and neck and is known for poor prognosis, and the majority of patients present with late-stage disease. Detecting the tumor early in the disease process ensures better treatment outcomes and prognosis. Chiang et al. showed that autofluorescence imaging is a useful adjunct used for screening for dysplasia and premalignant lesions in the oral cavity [34,35,36]. Noteworthily, the application of autofluorescence videoendoscopy for assessing the head and neck carcinoma is a promising method in the armamentarium of cancer management, because it has greater specificity and sensitivity. Gorpas et al. reported that oral lichen planus can be diagnosed by using a time-resolved fluorescence spectroscopy [37]. It is a noninvasive, time-efficient and safe procedure. The mucosa surrounding the tumor can be visualized effectively using autofluorescence (Figure 2). This means that the autofluorescence-based device can be used as an effective screening method and follow-up examination in a high-risk patient population.

The autofluorescence-based technique is a potentially novel procedure for screening and detecting oral malignant lesions early [38,39]. Any tissues that show fluorescent activity are potentially suspicious for malignancy, and hence should be properly biopsied. This technique, however, carries lower specificity, especially in the delineation and detection of oral squamous cell carcinoma [40]. 

Historically, microlaryngoscopy, which was first introduced by Kleinsasser in 1962, was the standard method for diagnosis and endoscopic treatment for precancerous lesions and early cancers of the larynx. However, this method is insufficient for accurate assessment of laryngeal lesions [28]. This can be partly due to factors such as limited view, difficult access, interpreter variability and suboptimal surgical theatre atmospheres. Recently, indirect autofluorescence endoscopy (AFE) has been applied in the comprehensive management of suspected precancerous and cancerous laryngeal lesions. Narrow-band imaging and autofluorescence endoscopy has been shown to be superior to white-light endoscopy in detecting the malignant mass [41,42,43]. There is also emergence of additional fluorescence staining for laryngeal neoplasms. This special staining is applied during routine endoscopic microlaryngoscopy, and it emerges as a promising adjunct diagnostic procedure for early identification of malignant neoplasm. These techniques are particularly efficacious in the assessment of precancerous and cancerous lesions [29,44]. The malignant mucosal alterations change from green fluorescence to reddish violet in appearance. The reddish violet AF signal becomes more intensive in the case of carcinoma in situ or severe dysplasia when compared with moderate dysplastic cases.

A single-blinded study by Roberto et al. that investigates the magnitude of neoangiogenesis in laryngeal and pharyngeal tumors showed that an enhanced contact endoscopy procedure allows clinicians to predict the histologic changes in laryngeal and pharyngeal lesions, either due to inflammation or cancer, based on fine evaluation of the characteristic neoangiogenetic changes in appearances [45]. Generally, these innovative procedures and instrumentations are regarded as critical tools that should be used in managing head and neck malignancy patients, especially those with upper aerodigestive tract tumor.

A further limitation of clinical examination by direct visualization is that, in inexpert hands, it would be difficult to differentiate between benign and malignant masses. This consequently may result in late patient referral to tertiary centers and, occasionally, it can also lead to overdiagnosis. The majority of premalignant lesions of the oral cavity are leukoplakia. Clinically, leukoplakia has the capacity to become malignant in about 5% of cases [46]. Importantly, the progression rate of dysplasia varies among the lesions, and even the in-charged specialist cannot predict which oral premalignant lesions have higher tendency to become malignant. Huang et al. used two-channel autofluorescence that highlights the NADH and FAD in the oral cancer and precancerous lesions and concluded that this technique is an effective method to delineate the presence of malignant lesions in the oral cavity [47]. 

Huang et al. conducted a study using VELscope autofluorescence in 47 oral cancer cases and 54 precancerous lesions, by using the intensity and heterogeneity changes produced by the lesions, and stated that the techniques can also effectively screen the malignant and premalignant lesions of the oral cavity [39,48,49,50,51]. On the other hand, a study by Ganga et al. failed to show the effectiveness of VELscope in detecting dysplastic lesions in the oral premalignant lesions [23]. The difference in results showed by this technique may be influenced by multiple factors, which may include interpersonal skills, observer bias and presence of concurrent inflammation at the tissues examined.

### 3.2. Diagnosis of Aerodigestive Tract Tumors by Autofluorescence Techniques

Head and neck cancer diagnosis requires a thorough clinical examination. A physical examination can determine an accurate clinical characteristic of tumoral mass, such as ulcerative pattern, infiltration depth or presence of multiple matted neck metastases. Endoscopic evaluation of suspicious malignant lesions with autofluorescence is mandatory to investigate all the subsites’ involvement to facilitate a comprehensive biopsy of selected tissues, which in turn can produce high representative tumoral tissues. In addition, these autofluorescent techniques will also allow clinicians to exclude the presence of second primary tumors. A complete biopsy of a suspicious lesion will produce accurate histological types of tumors, which is vital for a subsequent refined treatment plan.

**Figure 3:**
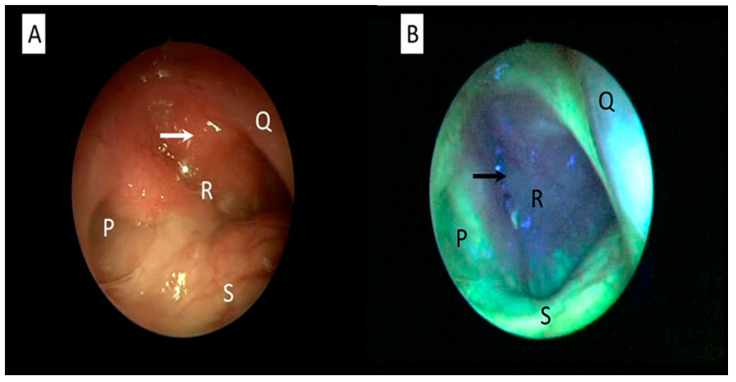
Nasopharyngeal examination of a patient with nasopharyngeal cancer using white-light (**A**) and autofluorescence mode (**B**). Abnormal mucosa around the cancerous area is clearly seen as autofluorescent loss compared with the normal healthy mucosa, which appears as cyan-green. An arrow indicates cancerous mucosa. Structure P: eustachian tube. Q: posterior part of nasal septum, R: nasopharynx, S: floor of nasal cavity (Figure is from author’s own collection).

During endoscopic autofluorescence evaluation, extra information about mucosal and submucosal changes to better characterize the lesion and its margins can be obtained. For instance, the nasopharyngeal area can be assessed with autofluorescence for any suspicious malignant changes (Figure 3). This technique allows early diagnosis of nasopharyngeal carcinoma [52]. A challenging issue is to determine whether conditions of chronic inflammation, such as chronic laryngitis and hyperkeratosis or pre-neoplastic lesions, such as leucoplakia, will show a mild or severe dysplasia [53]. The tissue biopsy and histopathological examination remain as the gold standard for diagnosis of any head and neck cancers, inclusive of oral cavity carcinoma [54]. In these cases, autofluorescent endoscopic techniques are useful for targeted tissue biopsy as they highlight the highly suspicious malignant areas. In select groups of patients, direct examination under general anesthesia with laryngoscopy, bronchoscopy and endoscopy will allow multiple biopsies from different anatomic subsites simultaneously. 

In a study by Ingrams et al. that evaluated the sensitivity, specificity and accuracy of autofluorescence in differentiating the benign versus the dysplastic and cancerous tissues in the oral cavity, they found out that the results were 90%, 91% and 91%, respectively [55]. Koch et al. in their study showed a higher sensitivity (97%) and specificity (95.8%) of using the VELscope for diagnosing OCSCC. The positive predictive value (PPV) was calculated as 41%, and the negative predictive value (NPV) was 75–80% [56]. On the other hand, Scheer et al., in their study using VELscope in diagnosing malignant oral lesions, documented that the sensitivity and specificity were 33.3% and 88.6%, respectively [57]. Another study found that autofluorescence was not highly specific for dysplasia and cancer, as fluorescence loss was observed in 87.5% of the benign oral lesions, leading to a low specificity of 12.5%. However, this device was unable to discriminate high-risk from low-risk lesions [58]. On the contrary, Luo et al. studied autofluorescence in the diagnosis of oral malignant lesions and compared it with digestive tract lesions, and it showed that autofluorescence has high accuracy and can be used effectively in managing head and neck malignancy [59]. It depends on the practicing clinicians to make a better choice of the instrument and tool to be used in managing the head and neck malignancy from arsenals of the available latest instruments.

### 3.3. Treatment of Aerodigestive Tract Tumors by Autofluorescence Techniques

The primary objective of tumor surgery is to maximize tumor removal and minimize the damage to the surrounding healthy tissue. Extensive resection with unnecessary removal of normal tissue may result in significant functional impairment, as the head and neck regions involve the structures that play vital roles in breathing, speech, swallowing and so forth. At the end of the spectrum, if the tumor resection is incomplete, the probability of the disease to recur is high. Recurrent tumors are difficult to manage and are associated with poor prognosis and survival. A positive surgical margin is associated with a dismal prognosis in terms of increased local recurrence and decreased patient overall survival [60,61]. Therefore, the ability to define the tumor resection margin with greater accuracy is a prerequisite in order to attain the best effective treatment for optimal patient management. For example, the usage of a fluorescence lifetime imaging technique in transoral robotic surgery enables the detection of oropharyngeal carcinoma in the setting of the management of metastatic carcinoma of unknown primary [62]. Additionally, the resection margin of oral and oropharyngeal cancer can be determined by using this technique [63,64]. This technique is an adjunct to visual inspection during intraoperative transoral robotic resection of oral cancers, in order to achieve a clear surgical margin [65].

Autofluorescent endoscopy aided with up-to-date scanning can further increase the effectiveness of surgical therapy of upper aerodigestive tract tumors. The developed scan with scanning time in the order of seconds can provide an objective, fast and cost-effective tool which is able to provide real-time assessment for a complete resection margin. Of note, this technique is able to quantify not only the surface mucosa but also different faces of the resection margins. In addition, Tajudeen et al. highlighted the usage of dynamic contrast imaging in aiding the good resection of tumor margins [66]. The availability of these various highly advance techniques will strengthen the management protocol for all head and neck cancer patients.

**Figure 4:**
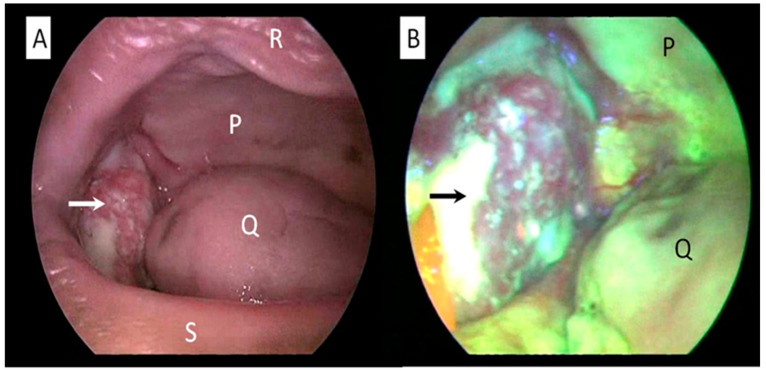
Oral cavity examination of a patient with oral cavity cancer using white-light (**A**) and autofluorescence mode (**B**). Abnormal mucosa around the cancerous area is clearly seen as autofluorescence loss compared with the surrounding normal healthy mucosa. An arrow indicates cancerous mass arising from buccal mucosa. Structure P: hard palate, Q: tongue, R: upper lip, S: lower lip (Figure is from author’s own collection).

To illustrate further, in the management oral cavity malignancy, the margin can be safely delineated by using autofluorescence (Figure 4). A safe resection margin is vital to avoid residual micrometastatic tumor deposits at the periphery of the main tumor. This is critical to hinder the tumor recurrence that is difficult to manage, as well as to prevent distant metastases. Leukoplakia should also be resected with a good margin in order to prevent a geographical miss. Leukoplakia, which is one of the important risk factors for oral cancer, may show hyperkeratosis with variable degree of dysplasia when examined microscopically [67,68]. Lesions at the dorsum of the tongue and floor of the mouth are the most probable to be dysplastic, especially in elderly patients who are chronic smokers. Erythroplakia is a rarer lesion but clinically significant, as it carries higher risk of malignancy [68]. Thus, meticulous assessment of erythroplakia is necessary, and biopsy is warranted in highly suspicious cases.

Multiple other head and neck surgeries can be revolutionized with the application of the current AF instruments that are widely available.

## 4. Advantages of Autofluorescence in Upper Aerodigestive Tract Tumor Management

Autofluorescence is easy to use, and it is a noninvasive method that can be combined with white-light examination techniques. This combined approach allows the acquisition of complementary information about mucosal tissue status. It helps the identification and selection of the lesions most at risk of degeneration and suspicious of malignancy. For instance, in laryngeal lesions, microscopic laryngoscopy in combination with AF can be used in general anesthesia during assessment of a suspicious laryngeal mass, lesion or leukoplakia. Kraft et al. documented that the autofluorescent endoscopy has higher sensitivity, specificity and accuracy than white-light endoscopy in the detection of premalignant and malignant lesions of the larynx [69]. Winiarski et al. showed in their study of forty patients that cancerous laryngeal mucosa showed lesser autofluorescent illumination [42], whereas Palasz et al. and Zargi et al. conducted a study in laryngeal cancer patients, and it showed a similar pattern of autofluorescent loss at the green spectra [70,71]. Additionally, Fostiropoulos et al. showed in their study of 152 patients with laryngeal lesions that autofluorescent imaging has a sensitivity of 98% vs. 88% and an accuracy of 97% vs. 90% compared with white-light endoscopy [72]. Imperatively, the application of this technique ensures negative surgical margins of laryngeal cancer surgery [73]. In contrast to other techniques such as photodynamic therapy, autofluorescence has several advantageous, namely it does not use photosensitizers, where multiple pharmacokinetics factors need to be considered during the application, and the prolonged preparation time associated with the photodynamic set up can also be avoided [40].

Noteworthily, with these autofluorescence techniques, surgeons are able to consider tumor margins in order to avoid radical resection, thus avoiding unnecessary damage to the normal surrounding healthy tissues. Halicek et al. and Fei et al. conducted a large study using hyperspectral and autofluorescent imaging to detect carcinoma intraoperatively and concluded that the techniques have multiple advantages, which include no radiation and no contact, and it has an increased rate of cancer detection compared with fresh-frozen sections [74,75]. Hyperspectral imaging is able to detect tissue absorption, scattering and fluorescence due to tumor tissue biochemical and morphological changes as a result of tumor invasion [75]. In addition, the usage of autofluorescence can minimize the requirement of unnecessary biopsies. These can save patient times and related morbidities [76]. Importantly, the autofluorescence-imaging examination can also be performed on patients who are medically compromised and are contraindicated for biopsies [77]. All of these pertinent advantages should be considered by the in-charge clinician and surgeon, so that better patient treatment can be corroborated.

At this juncture, there is a surge of scientific literature that focuses on the utilization of autofluorescence in the operation theatre in order to attain clear surgical margins during any cancer surgery cases [78,79,80]. Oncologically, clear surgical margins translate to better patient survival. Conventionally, clinical palpation, direct visualization and intraoperative ultrasound are used during surgery cases in order to obtain free resection margins. However, these techniques alone are limited, as sometimes hey can result in inadequate resection that requires re-excision, radical resection and sometimes overtreatment [81]. VElscope, for instance, has several advantages, as it is convenient to use, noninvasive, less time-consuming, able to provide real-time results and has no additional cost incurred [82]. Additionally, the VELscope can be used to detect resection margins of oral cavity carcinoma, which are not identified by conventional visual inspection due to its extended focal visualization length.

The study by Rashid et al. evaluated the prognostic benefit of using the VELscope to define the desired surgical margins during tumor resection which is oncologically safe [46]. Thirty-eight patients who had OCSCC underwent tumor resection at 10 mm bigger than the delineated margin by the VELscope, and they showed no local recurrence at 12 months post-surgery. Conversely, seven recurrences were noted in the control group, where conventional regular white light was used to delineate the OCSCC resection margin [54]. This finding favors the usage of AF during surgical resection of upper aerodigestive tract tumors.

The VELscope is a user-friendly camera device which is effective to be used for detections of mucosal changes the oral cavity based on the characteristic autofluorescence loss. It has been shown in many studies that the VELscope has high sensitivity in detecting oral malignant and premalignant lesions [57,83,84]. Another advantage of using the autofluorescent VELscope is that there is no special training necessary for both general and subspecialty practices. This device is a nonmagnifying device, which is used for direct visualization of oral mucosa changes. It is very simple to use and requires no technical measures such as the usage of dimmed light and reagents, and it is easy to assemble with less preparation time, which makes it a highly efficient device.

Recently, ViziLiteR PLUS, a modified version of the device, was introduced, in which a combination of autofluorescence and acidophilic blue dye are applied. This device allows the staining of the acidic substances in tissues such as DNA selectively. In the clinical setting, a one-minute rinse of solution containing acetic acid for desiccating oral tissue is performed, which is then followed by an oral examination taken with ViziLiteR PLUS with 430–580 nm wavelength light. Due to higher nuclear-to-cytoplasmic ratio present in the altered epithelial cells, the light reflected causes an aceto-white appearance of lesions in malignant cases. The normal cells, however, appear blue. In this first reported study, ViziLiteR was able to identify and detect a subclinical lesion, indicating occult epithelial abnormalities diagnosis [19]. This is a great significant benefit gained by using an AF assessment tool in the head and neck cancer management armamentarium.

For the D-LIGHT C/AF system that uses white-light endoscopy, which can be instantaneously switched over to autofluorescence endoscopy, it also possess several important clinical advantages. The procedure is highly effective, simple and safe to use, and it is easy to maneuver [83]. The diagnosis is made by autofluorescence techniques capable of producing strong positive results, and it may be offered as an excellent therapeutic adjunct to the conventional methods in the setting of tumor diagnosis of the upper aerodigestive tract system.

## 5. Disadvantages of Autofluorescence Techniques

At this juncture, autofluorescence endoscopic techniques have been applied not only at the clinic setting but also during surgeries in the operating theatre, as well as during investigational procedures in other clinical laboratories. Unfortunately, due to various obstacles, some clinicians and certain centers may have found difficulty in the usage and practice of AFI devices at their center. Such factors include an interobserver variability that limits AFI devices to clinicians with expertise, photodamage to biological material, low photostability of fluorophores and high background noise from autofluorescence from cytochromes and hemoglobin [77]. One of the other major limitations of intensity-based autofluorescence is its strong dependence on acquisition geometry. Acquisition geometry plays a critical role in obtaining accurate results from autofluorescent endoscopy procedures. For instance, changes in the working distance and angle of view can lead to totally different readouts, which, consequently, will yield false-positive or false-negative results. Thus, the clinician should be aware of this phenomenon and know how to handle the instrument and interpret the results with caution. Other disadvantages include high maintenance cost, insufficient expert assistant in handling the device and the false-positive-driven exogenous fluorophores.

Although autofluorescence imaging practically is cost-effective and noninvasive, it has an innate disadvantage, which is low specificity [17,22]. Several specific biomolecules and substances such as oxygenated hemoglobin, can greatly enhance the autofluorescence signal during endoscopy. False positives are observed in rich microvascularized tissues that cause scattering and autofluorescence loss, and these can be seen in inflammation, oedema and in granulation tissue. Additionally, in scenarios where there is massive hemorrhaging, the red blood cells able to absorb the excitation light, and it can significantly affect the evaluation of the tumorous lesions. In contrast, false negatives are observed in hyperkeratosis or in the areas where there are massive bacteria overgrowth. Selective bacteria are capable of producing extra fluorophores [84,85]. In fact, enormous bacterial spread and overgrowth stimulates and produces exogenous fluorophores, which include protoporphyrin IX. Protoporphyrin IX may influence the magnitude of the AF characteristics of the underlying mucosa in certain tissues. The production of these intrinsic fluorophores can greatly influence autofluorescence signals and may limit the application of AF endoscopy in clinical practice, for example, during an assessment of tongue carcinoma.

With regard to VELscope, some literature highlighted that, despite the good results attained, a primary limitation of the device when used by nonspecialists, sometimes, is it could lead to overdiagnosis. They emphasize that due to this limitation, examination by VELscope alone is insufficient for diagnosis and screening for potentially malignant disorders and OSCC compared with the conventional oral examination techniques. In addition, a study by McNamara et al. also suggested that the conventional visual examination, which includes systematic visualization and application of tactile sensation applied during vigorous inspection of the entire oral cavity, can be more effective than VELscope autofluorescence examination in a routine screening for any oral premalignant lesions [86,87].

Identafi is a mini-sized device. The Identafi probe is similar to a dental mirror, which can be easily maneuvered to visualize all tissues within the oral cavity and its anatomic subsites. Additionally, it is easy and flexible to handle compared with the VELscope. However, it has a limited applicability in general practice. It has been shown in a clinical trial, where the results of screening tests that used green, white and violet lights were compared with each other, that Identafi autofluorescence able to detect high-risk lesions but with limited tissue reflectance. Nowadays, clinicians are obtaining more data as compared with a conventional oral examination by using Identafi [46]. However, clinical training in the oral pathology and an optimal expertise are prerequisites; thus, its usage should be limited to selective tertiary referral centers with the availability of expert oral pathologists.

## 6. Future Prospect of Autofluorescence Endoscopy in Medical and Health Sciences

In the future, a combination of new technologies with existing techniques will be necessary to implement such technologies in the most cost-effective manner. The collaboration between surgeons, pathologists, radiologists, physicists and scientists is important to discover effective strategies in order to reveal the trajectory enhancement in patient care associated with these newly applied techniques. Hopefully, the autofluorescence-based techniques will be the hallmark in the near future for the early detection, assessment and comprehensive management of upper aerodigestive tract tumors. Of note, there is a possibility of introducing nanotechnology in autofluorescence imaging techniques. 

Nanotechnologies coupled with autofluorescence endoscopy could be an ideal evolution of AFI devices to simultaneously detect and diagnose oral pathologic lesions effectively in coming years [39,47]. The application of these techniques in general practice can escalate the positive therapeutic outcomes; hence, a refined and better cancer management can be achieved. 

Fluorescence lifetime imaging (FLIM) and two-photon imaging are both growing and are complementary techniques for elucidating, for instance, neural functions. However, combining these techniques together is complicated due to enzymatic binding, local viscosity differences, temperature, or pH changes in a biological milieu. Many fluorophores, both intrinsic and extrinsic, can exhibit multiple lifetimes and can change lifetimes depending on the local microenvironment [88]. Habibalahi et al. in their study of ocular surface squamous carcinoma used multispectral autofluorescence and applied artificial intelligence to map the cancerous margins [89]. They used a custom-built spectral imaging system with numerous different spectral channels. The machine learning method is used to detect the neoplasia margins based on the chemical composition changes that occur due to malignant transformation. Multispectral analysis showed that the cancer margin is similar to the margins in the stained pathology specimen. Fluorescent dyes have been developed and combined with antibodies or nanoparticles to function as contrast agents for molecular imaging by increasing the binding to the target site and providing more accurate information during tumor resection [78]. For instance, fluorescent gold nanoparticles can be used as a fluorescence-guided aptamer-targeted probe. This particle exhibits high water solubility, good biocompatibility and strong X-ray attenuation and can be used in computed tomography (CT) contrast enhancement. The study showed that fluorescent nanoparticle conjugates, used as molecular imaging agents to indicate the tumor location by CT imaging, may be easily observed in CT images with the naked eye [78]. Singha et al. reported that the development of reaction-based fluorescent probes is a highly intelligent innovation that is able to fulfil and enhance practical applications [90]. An effective probe with appropriate fluorophores depending on the tissue characteristics can be designed in order to gain the best outcomes for future usage in clinical practice. 

## 7. Conclusions

Early detection of upper aerodigestive tract tumors is crucial, as treatment strategy can be refined and started early for upper aerodigestive tract mucosal tumor patients. The emergence of autofluorescence imaging and endoscopy has improved the management of these tumors with resultant better patient prognoses and overall treatment outcomes. This technique has gained interest from many surgeons and clinicians around the globe. It can be used as a part of a mass screening of a high-risk population and for yielding an early and accurate histology diagnosis, as well as to enhance oncological resection of the tumors. This newer technique should be made available with less cost, not only to the major practicing institutions but also for patients at peripheries. Above all, the patient is the primary focus for the best care that humanity can provide and improve in order to have a viable, healthy and tumor-free future generation.

## Figures and Tables

**Table 1:** Types of Autofluorescence imaging techniques (AFI) that used in diseases and tumor detection.

	Type of AFI	Clinical Application	Characteristics
1.	DAFE system (Richard Wolf, Knittlingen, Germany)	Oral cavity, oropharynx and larynx cancers.	Xenon lamp that delivers blue light; a filter is inserted which changes from white to blue light examination or the reverse. Wu et al., 2018 [13]
2.	SAFE system (Pentax, Japan)	Laryngeal premalignant and cancerous lesions.	Combined video and autofluorescence Wu et al., 2018 [13]
3.	LIFE system (Xillix Technology, Canada),	Early diagnosis of lung cancer.	Endoscopic real image analysis Lam et al., 1998 [18].
4.	D-Light-AF system (Karl Storz, Germany)	Tumor diagnosis in the upper aerodigestive tract and laryngeal premalignant and cancerous lesions.	Blue light with Xenon arc lamp Wu et al., 2018 [13]
5.	VELscope LED Medical Diagnostics (Vancouver, BC, Canada)	Oral cavity, oropharynx and sinonasal cavity.	Noninvasive, easy handling and no training required Mascitti et al., 2018 [19] Lane et al., 2006 [20]
6.	Identafi DentalEZ (Lancaster, PA, USA)	Oral cavity, oropharynx and sinonasal cavity. Capable of finding new lesions not seen during COE. Used to compare tissue vascularity of potentially malignant disorders with the histological grading of the lesions using a vascular marker (CD34); it provides correlation between tissue reflectance and histological assessment of vascular structure, in both OSCC and noncancerous lesions.	Small size, more reliable than VELscope. Three kinds of lights—white light, green light and violet light Mascitti et al., 2018 [19]
7.	Others: (a) Sapphire Plus LD (DenMat Holdings, Lompoc, CA, USA), (b) DentLight DOETM Oral Exam System (DentLight, Richardson, TX, USA), (c) OralIDTM 2.0 (Forward Science Technologies, Stafford, TX, USA)	Oral cavity abnormalities and oral squamous cell carcinoma.	Autofluorescence-based Kahn, et al., 2018 [21]

## Data Availability

Not applicable.
